# Chemical Facets of Superconductivity in UTe_2_

**DOI:** 10.1021/jacs.5c12203

**Published:** 2025-09-18

**Authors:** Eteri Svanidze, Andreas Leithe-Jasper, Marcus Schmidt, Nazar Zaremba, Mitja Krnel, Yurii Prots, Ulrich Burkhardt, Markus König, Reiner Ramlau, Berit H. Goodge, Yuri Grin

**Affiliations:** 28270 Max Planck Institute for Chemical Physics of Solids, Nöthnitzer Str. 40, Dresden 01187, Germany

## Abstract

Understanding superconductivity requires a deep comprehension of the chemical structure. The discovery of a new unconventional superconductor UTe_2_ a few years ago prompted many detailed investigations of its physical properties. Despite its unconventional ground state being rather well-studied, a strong sample-to-sample variation of superconducting behavior as a result of different preparation conditions has remained largely unexplained until now. In this work, an in-depth analysis of the UTe_2_ crystal structure and resultant physical properties by implementing several types of synthetic routes was carried out. The difference between superconducting and non-superconducting UTe_2_ lies in the presence of uranium vacancies, on the order of 4%. As a result, the *b* and *c* lattice parameters vary, yielding a volume difference of about 0.51%. A subtler difference between samples exhibiting one and two superconducting transitions is driven by local deviations from the translational symmetry in the main atomic arrangement. Several well-pronounced maxima have been observed in the difference density map, predominantly located in the *bc* plane due to a local appearance of the similar atomic arrangements in different orientations. The extreme sensitivity of UTe_2_ to such a small number of defects re-emphasizes the unconventional nature of superconductivity in this compound. Furthermore, this work underscores the importance of a thorough, combined chemical and physical analysis of intriguing strongly correlated materials—in particular for compounds that are known to exhibit nontrivial ground states and exotic accompanying phenomena.

## Graphical abstract

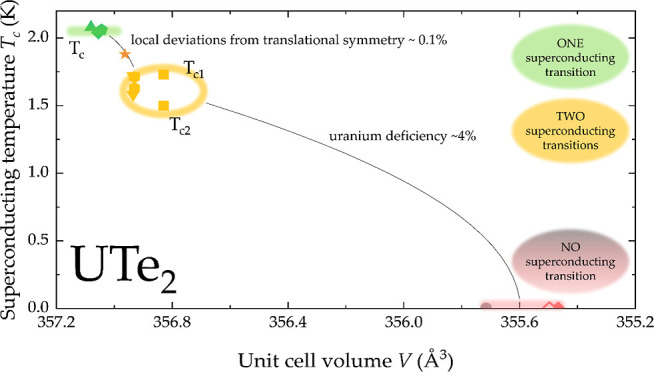

## Introduction

A renaissance of uranium research in condensed matter physics and solid-state chemistry was brought on by the discovery of an unconventional superconductor UTe_2_, with nearly 300 papers focusing on this material since the first report of superconductivity nearly five years ago.^1^ While uranium-based superconductors are sparse—less than two dozen compounds show superconductivity without tuning (see Figure 1)—nearly half of the known systems have unconventional superconducting pairing mechanisms, with several spin-triplet candidates.^2–10^ Uranium-based superconductors display a multitude of peculiar phenomena:^11^ coexistence of superconductivity and magnetism,^12–14^ the so-called “hidden order”,^15–17^ enhancement of effective electron masses,^3,9,18^ field-induced superconductivity,^19–21^ and peculiar topological features.^1,22–24^

**Figure 1:**
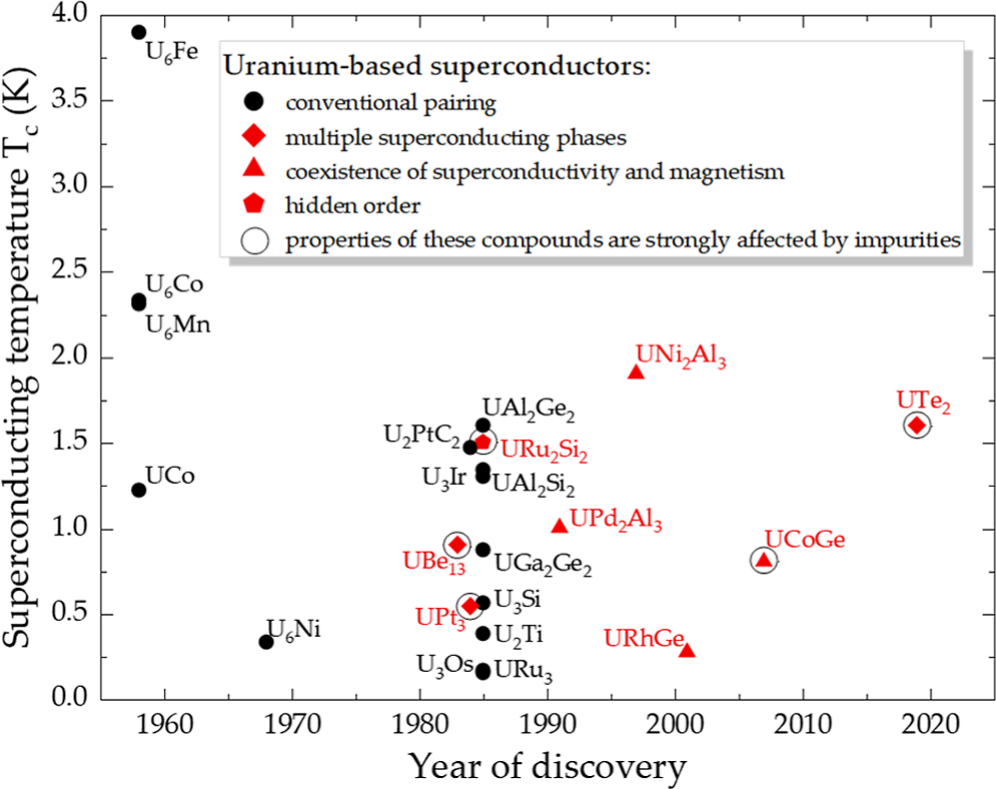
Timeline of the research on uranium-based superconductors. A significant portion of these (indicated by red symbols) are unconventional and show unique accompanying phenomena. In several systems (circled in black), even minor crystallographic imperfections (on the order of 1–2% at.) have a pronounced impact on their physical properties.

Due to the small energy scale of uranium-based strongly correlated compounds, the quest for crystals as close to perfection as possible is of utmost importance; exciting phenomena can be hidden if the crystal quality is not sufficiently high. On the other hand, increasing the size of crystals may enhance the probability of the structural inhomogeneities. In general, robustness against impurity effects was suggested to hint that superconductivity is likely conventional in nature.^10,25–33^ Since theoretical analyses of solid-state materials often assume an ideal crystal structure, knowing the precise composition and atomic arrangement is essential. This information is crucial not only for understanding the system’s properties but also in controlling them. So far, several of the unconventional uranium-based superconductors—marked by black circles in Figure 1—have been suggested to have crystallographic defects, for example, stacking faults in UPt_3_,^34–36^ Al inclusions and vacancies in UBe_13_ originating from the flux preparation,^37–39^ and spatial inhomogeneities of URu_2_Si_2_.^40–44^ Unveiling the chemical complexity of UTe_2_ is the main topic of this work.

From a physicist’s perspective, the value of the residual resistivity ratio (RRR) is often used to describe the quality of the crystal. Defined as the ratio of resistivity at room temperature to that at the lowest measured temperature, a higher number typically signals a better crystal quality. This metric is based on Matthiessen’s rule, which states that electrical resistivities in metals are additive, arising from the sources such as electrons, phonons, and impurities. For most materials, the RRR value can be estimated from the zero-limit resistivity, assuming that the room temperature resistivity is impurity-independent. For example, the RRR for some elements of exceptional purity can be above 30,000 (6N or 99.9999% Cu).^45^ For solid-state compounds, a value of 500 corresponds to ∼100s ppm impurity levels,^46^ which is typically below the resolution achievable through a comprehensive chemical analysis. The reason(s) behind the chemical origin of “imperfections”, however, and their impact on the value of RRR are not always clear.^47^ Furthermore, it is important to note that many strongly correlated materials are not simple metals, from the point of view of chemical bonding. Moreover, for superconductors, it is common to take the value right before their entrance into superconducting states, which is not always well-defined. For this and other reasons, the RRR is not always the most reliable metric for assessing crystallographic perfection.

In general, RRR values on the order of 100 are thought to indicate good sample quality, with respect to its transport properties, which can in turn be confirmed by single crystal X-ray diffraction (global probe), electron-dispersive X-ray analysis (global or mesoscopic probe), transmission electron microscopy (local probe), and inductively coupled plasma spectroscopy (global probe). Perpetual advancements of synthesis techniques over the recent decades have been crucial in realization of the intriguing quantum phenomena in solid-state materials, giving us access to single crystals of unprecedented quality.^48–53^ For congruently melting uranium-based compounds, growth by means of a floating zone or a Bridgman technique can often yield RRR values over 800, as illustrated by the cases of URu_2_Si_2_ ^42,43^ and UPt_3_.^42,54,55^ Unfortunately, UTe_2_ is not a congruently melting material. It also seems to have a compositional homogeneity range,^56–59^ coupled with high vapor pressure of tellurium, making flux or chemical vapor transport (CVT) synthesis more suitable. To date, single crystals of UTe_2_ has been grown via tellurium flux (RRR ∼3),^1^ chemical vapor transport (RRR ∼30–90),^58,60^ salt flux (as high as RRR ∼1000),^50,61,62^ and hybrid CVT-salt-flux (RRR ∼1000) methods.^50^ Overall, the salt flux and hybrid CVT-salt-flux methods typically result in samples with less sample-to-sample variation, compared to the CVT-grown samples, as we also show for our samples in Figure S4. Even though slight air sensitivity of UTe_2_ ^60,62,63^ suggests that values of RRR must be taken with caution, it is clear that there should be an underlying chemical difference between UTe_2_ grown by different methods. Indeed, samples with low RRR typically do not exhibit superconductivity, unless exceptionally high magnetic fields are applied.^64^ Some of the previous studies have suggested that single crystals of UTe_2_ are prone to a number of crystallographic imperfections such as spatial inhomogeneities,^58,59,65^ uranium deficiencies,^56,58^ and tellurium vacancies.^62^ In this article, we examine all of the previously put forth hypothesis and propose a new type of structural inhomogeneity that has not yet been reported for UTe_2_.

## Results and Discussion

In an effort to conclusively establish the intrinsic crystal structure and the influence it has on the superconducting properties of UTe_2_, several batches of this compound were prepared, employing the same recipes used in the previous work—see Table S1 for details.^1,57,59,60,62,65^ In addition to the aforementioned CVT and salt flux methods, we carried out an oxygen-free[Fn fn1] synthesis of UTe_2_ (see the Supporting Information). Furthermore, another batch was synthesized using two Te sources: in addition to elemental Te, a small amount of TeO_2_ was used to add a specific amount of oxygen to the system. The specific heat of several crystals was then measured in order to determine superconducting properties; note that for CVT-grown samples, differences exist from one crystal to another (see Figure S4). Consequently, microscale specimens for single crystal diffraction were extracted from the same crystals; see Figure 2. This meticulous approach ensures that we capture the intrinsic relation between the chemistry and physics of UTe_2_ prepared by various methods. Similar to previous reports, all UTe_2_ samples can be grouped in three categories: (i) non-superconducting (red and gray, Figure 3, top panel), (ii) superconducting with two transitions (yellow, Figure 3, top panel), and (iii) superconducting with one transition (green and orange, Figure 3, top panel). Similar to previous reports,^59^ we find that samples with higher *T*_c_ only show one superconducting transition. For the sample with the sharpest transition from the current study (Sample 8, *T*_c_ = 2.04 K, Figure 3, bottom panel), the specific heat parameters are consistent with what has been observed for the current “state-of-the-art” UTe_2_ samples (Δ*C*_e_/(γ*T*_c_) = 2.6 and γ_0_/γ_N_ = 0.034 ^50^ vs Δ*C*_e_/(γ*T*_c_) = 2.2 and γ_SC_/γ_N_ = 0.089 for our sample shown in Figure 3).

**Figure 2:**
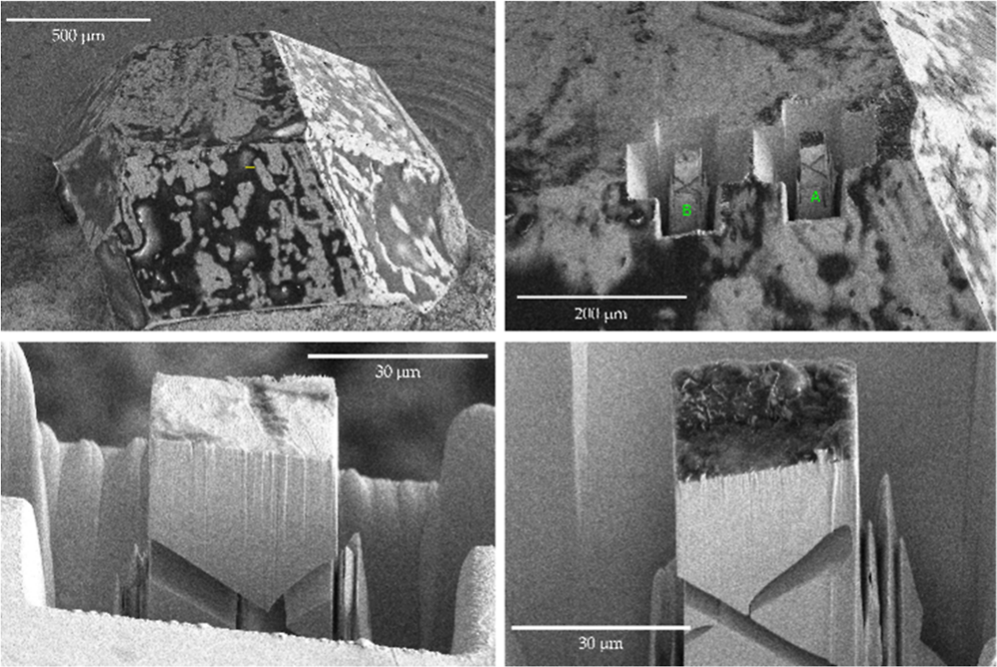
A UTe_2_ single crystal (top left) was used to measure specific heat data. Specimens for single crystal X-ray diffraction experiments were isolated with a focused ion-beam technique (top right) from the exact crystals used for specific heat measurements. The resultant cuboids (bottom left and right) typically have the size on the order of 30 × 30 × 30 μm.

**Figure 3:**
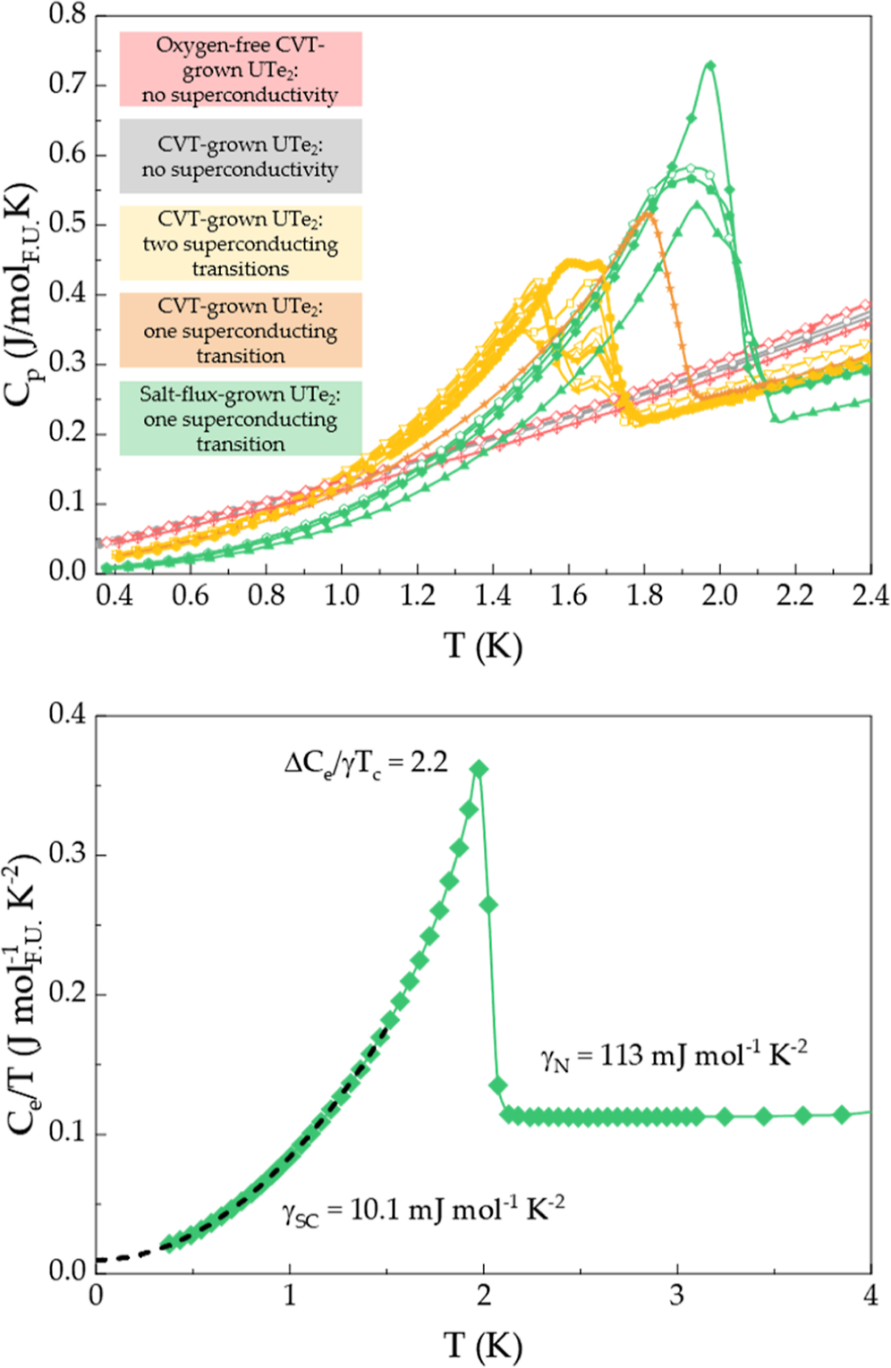
Superconductivity in UTe_2_: Specific heat of several UTe_2_ samples prepared in this work (top). In addition to non-superconducting samples (gray and red), samples with one (green) and two transitions (yellow) are investigated. For the sample with the sharpest transition and highest *T*_c_, extracted parameters are comparable with what has been reported for the most recent “state-of-the-art” samples (bottom).

It is important to note that a precise estimate of the unit cell parameters obtained from powder X-ray diffraction data with a standard can reflect the corresponding change of the physical properties—such as, for example, the value of superconducting critical temperature *T*_c_.^47^ However, prior to this work, the vast majority of the structural investigations on UTe_2_ have focused on single crystal data,^58,59,65^ with the reports on powder X-ray diffraction being rather limited.^66–69^ Moreover, for the latter, a concurrent investigation of superconducting features was not carried out as most of these investigations were performed prior to UTe_2_ being identified as a superconductor. As summarized in Figure 4, the lattice parameters of UTe_2_ are rather similar for various batches. However, a closer look indicates that there is a clear difference—especially in the values for [010] and [001]. This results in an overall change of the unit cell volume of 0.5% between non-superconducting (red and gray) and superconducting (yellow, orange, and green) samples. The response of superconductivity to such remarkably small lattice changes in UTe_2_ supports its unconventional pairing mechanism.^31–33^ Interestingly, another uranium-based superconductor UBe_13_ displays similar sensitivity to minute changes in the crystallographic lattice—in U_1–*x*_Th_*x*_Be_13_, the superconducting transition is first suppressed from 0.9 to 0.4 K (corresponding lattice change of 0.02%) and then split into two superconducting transitions (corresponding change of 0.04%).^70–72^ In UTe_2_, the difference in the unit cell volume between the sample with one and two superconducting transitions is only 0.1%—far below the resolution limit of most solid-state characterization techniques (see the discussion above).^47^ However, it must be pointed out that the loss of superconductivity in UTe_2_ is not only due to the change in volume—hydrostatic pressure^69,73–75^ also destroys superconductivity, but the corresponding change in the volume is on the order of 4% (∼1.5 GPa). Furthermore, strain investigations indicate that superconductivity is suppressed when strain is applied along the [100] and [010] directions, while strain along the [001] direction increases the value of *T*_c_.^76,77^ We assume that this volume change is reflected in the change in the uranium oxidation state. As shown previously, the oxidation state of uranium in UTe_2_ is close to 3.3–3.7 at ambient conditions.^78–80^ Since we see a reduced volume of non-superconducting samples, this is consistent with a shift of the uranium oxidation state from more 3+ toward more 4+. As shown in ref 81, even small changes in pressure result in a significant change of the uranium electronic state in UTe_2_. This means that the specific electronic state of uranium is just as delicate as it is crucial for the appearance of superconductivity in UTe_2_.

**Figure 4:**
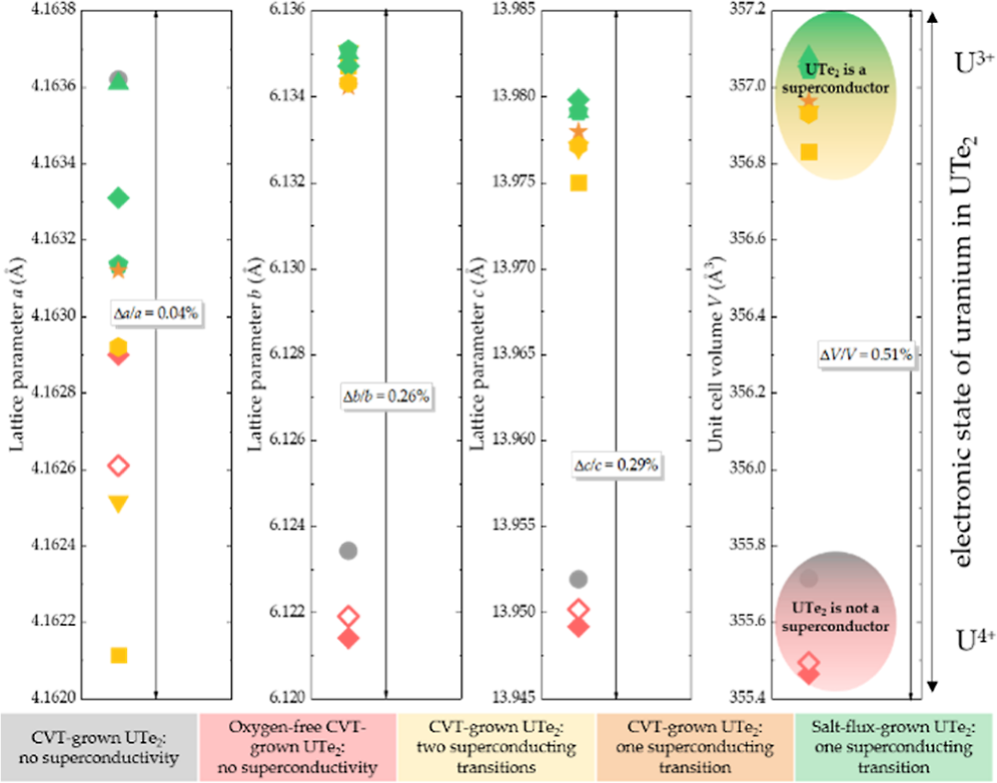
Lattice parameters of UTe_2_ show a clear difference between samples prepared by different means—if the values are extracted from the powder X-ray diffraction data, performed with a standard. In particular, a change of the unit cell volume on the order of 0.5% is evident between the samples that do (green, orange, and yellow) and do not (red and gray) show superconductivity. There is also a small, albeit discernible unit cell volume change between samples showing one and two superconducting transitions. It is likely that this change in volume is reflecting the change in the oxidation state of uranium.

In order to get insight into the chemical origin of the difference in superconductivity of UTe_2_, we examine single crystal diffraction data measured on nine single crystals prepared by different techniques:

- Two non-superconducting samples: oxygen-free CVT (Sample 1) and traditional CVT (Sample 2);
- Three samples with two superconducting transitions: CVT in quartz tubes (Samples 3 and 4) and a glassy carbon crucible (Sample 5);
- Four samples with one superconducting transition: CVT (Sample 6) and molten-salt flux (Samples 7, 8, and 9).

The details of single-crystal diffraction experiments and structure refinements are summarized in Tables S2 and S3. Similar residual factors and numbers of reflections used for refinement allow direct comparison of the obtained results. For all nine crystals, the refinement of the atomic coordinates and displacement parameters converges without problems and yields low residual values between 0.024 and 0.034, resembling previous results.^56^ The first characteristic result of the structure refinements is the presence of ∼4% defects on the uranium site in the single crystals that do not superconduct; this correlates with the reduced values of the lattice parameters and, as a result, unit cell volume for these crystals (Figure 4, gray and red symbols). It is important to note that reduced occupancy of U by a similar amount was previously observed in non-superconducting UTe_2_.^56^

The structure and quality of two representative UTe_2_ samples were investigated by atomic-resolution scanning transmission electron microscopy (STEM). In particular, we have examined Sample 1 (non-superconducting, oxygen-free CVT) and Sample 5 (two superconducting transitions, traditional CVT). Figure 5 (top) shows representative atomic-resolution high-angle annular dark-field (HAADF)-STEM images of Sample 1 along the [100] projection (left) and Sample 5 along the [010] and [110] projections (middle and right, respectively). Both, in general, show excellent adherence to the expected crystalline structure. Certain atomic columns of the uranium sites, as marked by the yellow arrows, show reduced intensity, which would be consistent with uranium vacancies, as shown from the X-ray diffraction. The red arrows highlight regions that could suggest subtle distortions in the lattice. Larger fields of view, such as the one shown in Figure 5 (bottom), are sampled using low-angle annular dark-field (LAADF)-TEM, which is more sensitive to diffraction contrast arising, e.g., at local strain fields near defects in the crystal.^81–83^ We find no features that could be suggestive of more extended defects (e.g., stacking faults) in either samples within the accessible areas of the STEM lamellae—on the order of 10^8^ Å^2^. This suggests that the length scale of these local structural inhomogeneities is on the order of a few unit cells.

**Figure 5:**
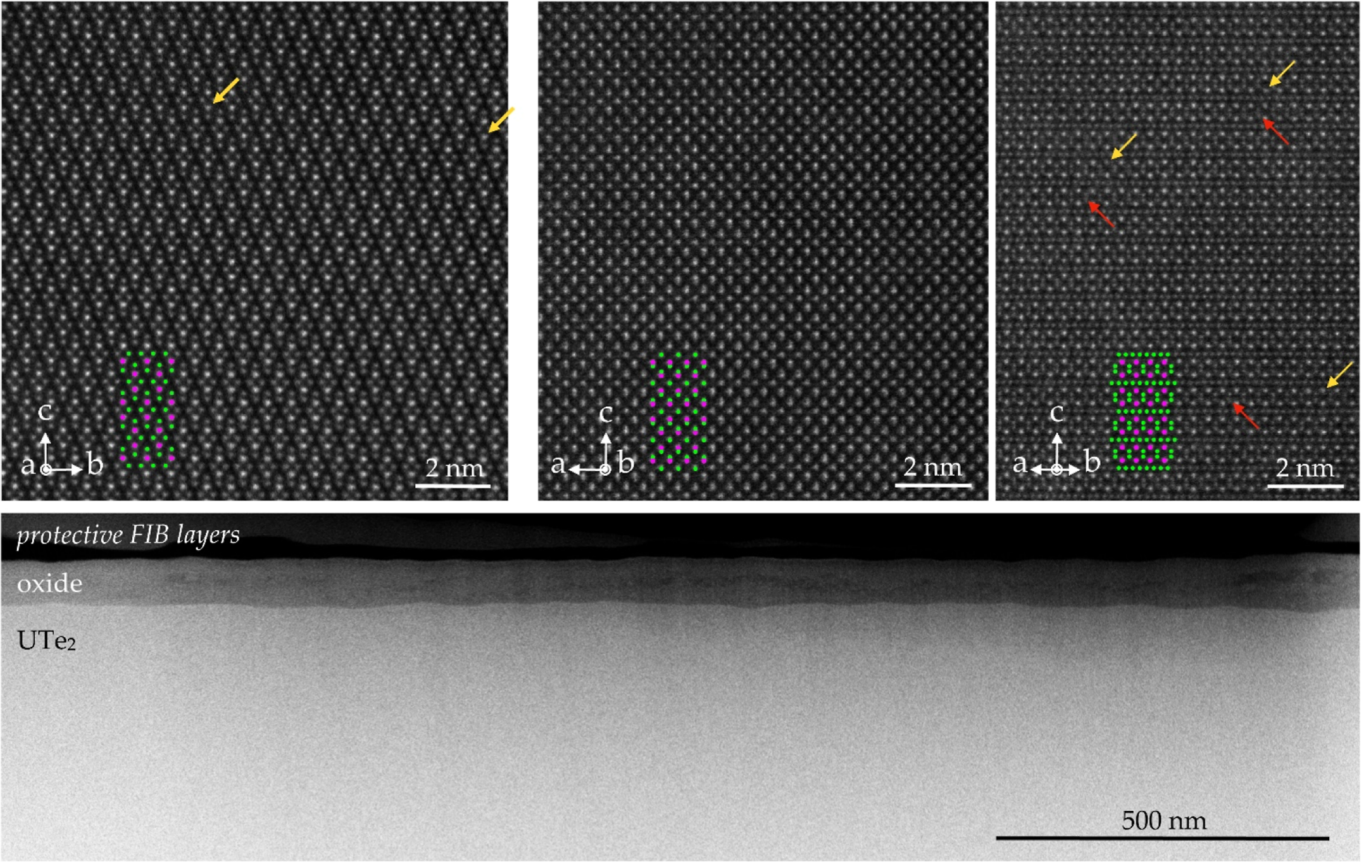
Top: Atomic-resolution high-angle annular dark-field (HAADF)-STEM images of Sample 1 (oxygen-free CVT, non-superconducting) along the [100] projection (left) and Sample 5 (regular CVT, two superconducting transitions) along the [010] and [110] projections (middle and right, respectively). A low-angle annular dark-field (LAADF)-STEM image of a larger area for Sample 1 (oxygen-free CVT, non-superconducting). Yellow arrows indicate uranium columns with reduced intensity, possibly related to vacancies, observed in the X-ray diffraction data. Red arrows highlight possible distortions in tellurium columns.

An analysis of the final residual electron density maps after the refinement of the ordered crystal structure of UTe_2_ (space group *Immm*, one U and two Te positions) reveals that, independent of the preparation conditions, all crystals show common behavior that can be directly linked to the respective superconducting properties. The final difference density maps are not, as expected, featureless, but rather show well-pronounced maxima at the same positions (for example, in the (100) plane, see Figure S3). Characteristic are the maxima on the lines [00*z*], [01/2*z*], and [0 ≈1*z*] (Figures 6 (top) and S3). Accounting for these positions in the form of additional sites during the refinement reduces the residual value slightly.

**Figure 6:**
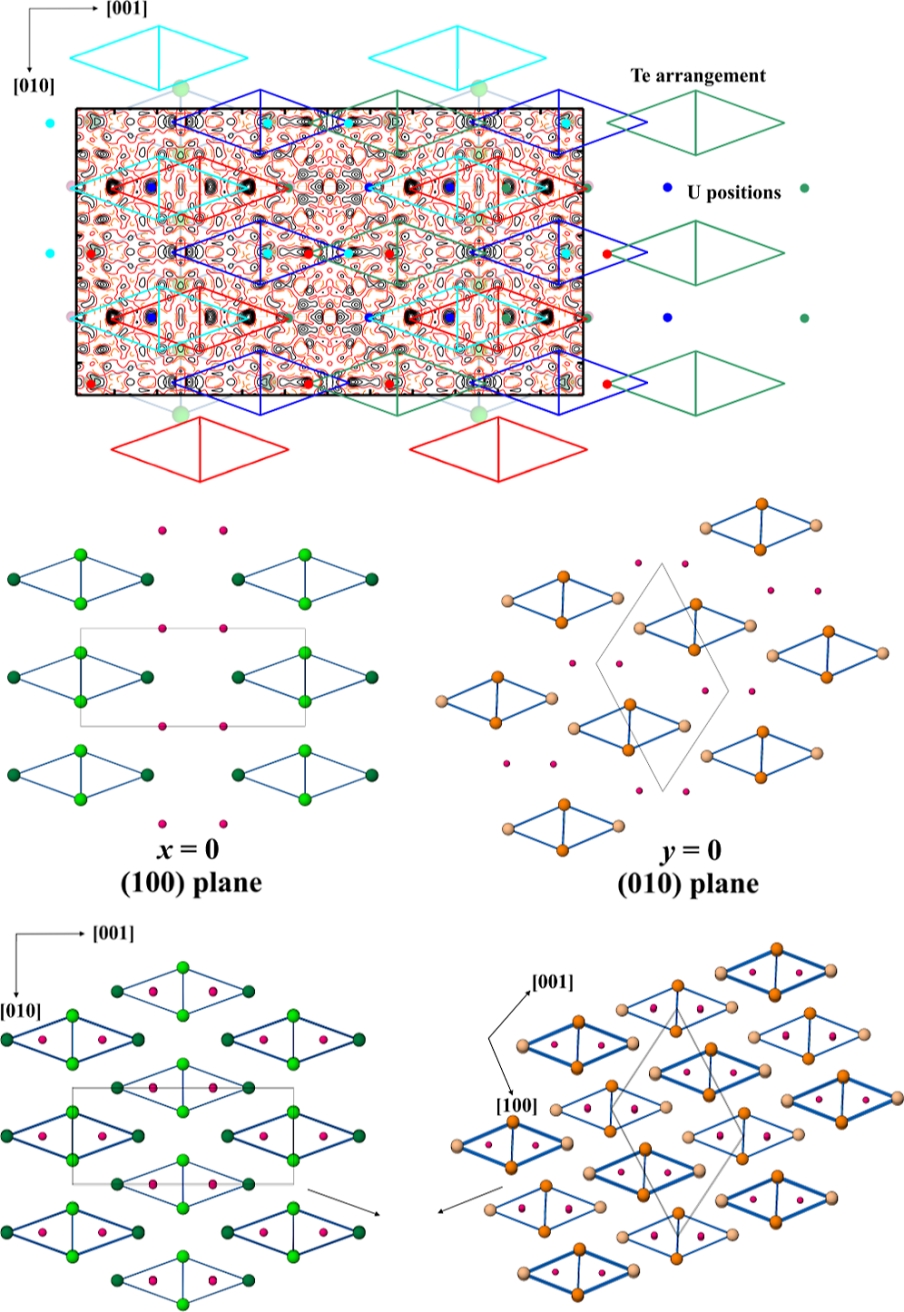
Difference electron density map in the (100) plane of UTe_2_ (Sample 5, two superconducting transitions) after the refinement of the ordered crystal structure showing the presence of four additional atomic motifs in different orientations (top). Green, red, cyan, and blue colors represent four different components that are still UTe_2_ but are shifted with respect to each other. Atomic arrangements in the (100) and (010) planes of the structures of UTe_2_ and NbAs_2_ types, respectively (middle). Crystal structures of orthorhombic UTe_2_ (space group *Immm*) and monoclinic NbAs_2_ (space group *C*2/*m*) types with similar arrangements of the rhombic prisms around transition metal “dumbbells” (bottom).

Assuming an occupancy of the most characteristic position at (00 ≈0.23) results in a distance of ∼1.9 Å to the next uranium atom and ∼2.1 Å to the next Te position, and the distance from the position at (01/2 ≈0.08) to the next Te atom is about 1.7 Å. These distances are short, but similar values were reported for γ-UO_3_ (1.80 Å)^84^ or β-UO_3_ (1.79, 1.89, 1.98 Å).^85,86^ A slightly larger distance of ∼2 Å have been seen in other uranium oxides.^85^ Characteristic Te–O distances are typically between 1.8 and 2.0 Å.^66^ Therefore, the occupancy of both positions by oxygen was the initial hypothesis of this work. This also makes sense given that the majority of UTe_2_ samples prior to this work have been made in quartz tubes, which provide a convenient source of oxygen during the growth. However, a detailed analysis of Samples 1 and 5 with the help of electron energy loss spectroscopy (EELS) did not reveal the presence of oxygen in the bulk on the atomic level (some oxygen was observed on the surface due to high air sensitivity of UTe_2_). Moreover, preparation of UTe_2_ using TeO_2_ as one of the Te sources did not result in a marked “occupancy” increase for the two aforementioned positions (Sample 3, prepared with extra added oxygen). Furthermore, the sample prepared in a glassy carbon crucible (Sample 1)—which eliminates the majority of oxygen—did not yield an essential reduction of the “occupancies” of these positions. Thus, the incorporation of oxygen into the UTe_2_ lattice cannot be considered as an essential chemical reason for the emergence of superconductivity.

On the other hand, there must be an underlying chemical difference between samples of UTe_2_ that show one and two superconducting transitions (green, orange, and yellow in Figure 3). The difference in volume is small but non-negligible, on the order of 0.1%, while the occupancy of uranium is basically 100%. The residual density map (Figure 6) suggests the presence of local deviations from the translational symmetry of the main atomic arrangement, i.e., a local appearance of another atomic arrangement or the same atomic arrangement with a different orientation. This was previously shown to be the case for the *R*AlB_4_ (*R* = Tm, Er, Yb^87–89^ ) compounds and recently for Be_2_Ru^90^ as well UFe_5_As_3_.^91^ In such a case, the minority atomic arrangement is reflected in the appearance of clearly structured maxima in the difference electron density map. When the density of such defects is sufficiently high, they can be observed by STEM imaging.^89,90,92,93^ For the case of UTe_2_, we estimate from the X-ray single crystal diffraction data that this type of defect is present on the order of 0.1%. This explains the lack of a clear signature in STEM, except for some small local features, as summarized in Figure 5.

The residual electron density calculated for UTe_2_ shows that the sample showing two superconducting transitions (Sample 5) may be understood as a superposition of not just two but four (!) similar lattices (Figure 6). The basic structural motif of all four components is resembling the UTe_2_ one^66^— see, for example, the atomic arrangement of the (100) plane shown in Figure 6 (middle left panel). The latter can be represented as a packing of side face-centered rhombic prisms (or double triangular prisms) formed by tellurium atoms around the dumbbells of uranium species (Figure 6, bottom left panel). A similar building principle is present in the crystal structure of NbAs_2_ (Figure 6, bottom right^94,95^ ). The difference between both atomic arrangements is in the relative orientation of the rhombic prisms. They are shifted from one layer to another along the directions marked by arrows in Figure 6 (bottom panel). The relative positions of the four-atom rhombic Te arrangements (bases of the rhombic prisms) to each other in the (100) plane (red vs green or blue vs magenta in Figure 6, top) resemble the relation of atomic arrangements in the NbAs_2_-type structure (Figure 6, middle and bottom right). This means that the translational symmetry in the structural matrix of UTe_2_ is locally violated and other atomic arrangements may appear, similar to the cases of *R*AlB_4_, UFe_5_As_3_, and Be_2_Ru described above. Besides the main motif of UTe_2_, there seems to exist at least two others. The first one is similar to the basic one, but shifted along [001] with respect to the latter. The second one may be similar to that of NbAs_2_. From the comparison of the residual density maps (Figure S3), the amounts of such defects vary between UTe_2_ prepared in different ways—this is consistent with what has been seen in other tellurides of the rare-earth metals.^95,96^

Taking electronic instability as a starting point for the appearance of superconductivity, the chemical peculiarities of UTe_2_ and their role in superconductivity can be understood within the following scenario. If the stoichiometric composition reflects a stable electronic state of U, then the deficiency of the U position will change this. The accompanying reduction in volume is on the order of 0.5%, which destroys superconductivity of UTe_2_ (green vs red symbols in Figure 4). If in some parts of the structure, the UTe_2_-type pattern is transformed into the NbAs_2_-type one, the nearest environment of the uranium atoms changes: in a UTe_2_ pattern, there are 4 (0b)Te and 4 (1b)Te (dark- and light-green, respectively, in Figure 6 bottom left) around U, while in the NbAs_2_ pattern, there are 3 (0b)Te and 5 (1b)Te (light- and dark-orange, respectively, in Figure 6 bottom, right). This, in turn, changes the electronic states of uranium even further and gives a small, albeit perceptible, change in volume on the order of 0.1% (yellow vs green symbols in Figure 4). These small defects result in a global change of the uranium electronic state, splitting superconducting transition into two. For UTe_2_ specifically, the uranium oxidation state at ambient pressure has been estimated^78–80^ to be approximately 3.3–3.7+, trending toward 4+ under increasing pressure.[Fn fn2] It is plausible that uranium vacancies replicate this effect by reducing the unit cell volume and pushing the oxidation state closer to 4+. On the other hand, the emergence of a second superconducting transition also appears to be oxidation-state-driven, likely caused by local violations of translational symmetry. We propose that this mechanism is analogous to what has been observed in UPt_3_, where stacking faults lead to similar phenomena.^34,97,98^ To further explore the latter, a detailed investigation of UPt_3_ using advanced techniques is warranted, work that is currently underway.

## Conclusion

In summary, two types of crystallographic imperfections play an essential role in the appearance of superconductivity in UTe_2_.[Fn fn3] The first type of pore is the partial occupancy of the U position, which was already partially discussed by previous reports. The second one—a local formation of atomic arrangements, different to the basic matrix—was established as part of this work. The discovery of the latter was only possible via an in-depth analysis of the residual electron density maps obtained from single-crystal X-ray diffraction combined with the high-resolution TEM study. Both types of crystallographic imperfections likely change the oxidation state of uranium in UTe_2_. The diverse spectrum of superconducting behaviors of UTe_2_ can therefore be interpreted as an interplay of two structural peculiarities.

## Supplementary Material



## Footnotes

a The term oxygen-free refers to the fact that the sample does not come in direct contact with the quartz ampule since the material is placed inside a glassy carbon crucible. And while the sample is sealed under argon, a very small (∼few ppm) amount of oxygen is possibly still present in the sample container during synthesis.

b It is important to note that the room temperature value of the uranium oxidation state in UTe_2_ in refs 78–80 has been estimated on the CVT-grown samples. We suspect that the value for the MSF-grown samples is similar but not exactly the same, suggesting that this type of spectroscopic investigations would be of great interest.

c In order to definitively decouple the respective impact of these crystallographic imperfections on the superconductivity of UTe_2_, electron irradiation experiments would be of interest—in particular, the question is whether or not the single superconducting transition can be split into two.

## Notes

Further details regarding synthesis, as well as chemical and physical analyses (PDF)
