# Supplementary material for: Chemical Facets of Superconductivity in UTe2
Source: J Am Chem Soc. 2025 Sep 18;147(39):35809–17. doi: 10.1021/jacs.5c12203 (PMC12498385; doi:10.1021/jacs.5c12203)
Supplement: Supplementary file 1 [file ja5c12203_si_001.pdf]

Chemical facets of superconductivity in UTe_2

Eteri Svanidze*, Andreas Leithe-Jasper, Marcus Schmidt, Nazar Zaremba, Mitja Krnel, Yurii Prots, Ulrich Burkhardt, Markus König, Reiner Ramlau, Berit Hansen Goodge, Yuri Grin

Max Planck Institute for Chemical Physics of Solids, Nöthnitzer Str. 40, 01187 Dresden, Germany

SUPPORTING INFORMATION

Methods:

Synthesis: Given slight air-sensitivity of UTe_2, coupled with its mild toxicity, all sample preparation and handling took place in a dedicated glove box system (O_2, H_2O < 0.1 ppm).[1,2] In order to examine the relationship between crystallographic properties and resultant superconductivity (or lack of thereof), we have prepared single crystals of UTe_2 in three different ways – oxygen-free CVT, traditional chemical vapor transport (CVT), and salt flux. As mentioned in the main text, by oxygen-free synthesis, we mean that the contact between the sample and the quartz tube (a significant source of oxygen) is avoided when glassy carbon crucible is used. Of course, miniscule amount of oxygen (~ a few PPM) could still be present in the ampoule, and, consequently, in the sample. However, in-depth TEM analysis did not indicate presence of oxygen in our UTe_2 samples. While the first and third synthesis methods are currently used by other groups, this report is the first in which oxygen-free CVT was implemented. All methods yielded mm-sized crystals, with some images shown in Figure S1:

1. Oxygen-free CVT (Sample 1):

- First, uranium powder was fabricated by hydrogen decrepitation technique[3] treating uranium wire (natural uranium, Goodfellow, 99.9 %) under an atmosphere of Ar/H_2 (90/10 vol%) between 200°C and 400°C within an alumina crucible placed in a quartz ampoule connected to a gas reservoir. Several heating and evacuating (dynamic vacuum) cycles were applied resulting in a fine U-powder.
- The quartz ampoules as well as glassy carbon crucibles (Figure S1) were heat treated under dynamic vacuum prior to use.
- Tellurium (Alfa Aesar 99.999%) was powdered in a glove box prior to use.
- Iodine (Alfa Aesar 99.998%) was placed directly into the glassy-carbon crucible.
- The quartz ampoules were loaded in a glove box (O_2, H_2O < 0.1 ppm), sealed under dynamic vacuum (10^{-4} mbar) and placed in a furnace with a variable temperature gradient.
- UTe_2 crystallized by a chemical transport reaction in a temperature gradient with the mixture of the starting materials at the hot source and the formed crystals at the cool sink.

2. Traditional CVT (Samples 2, 3, 4, 5, and 6):

- First, uranium powder was fabricated by hydrogen decrepitation technique[3] treating uranium wire (natural uranium, Goodfellow, 99.9 %) under an atmosphere of Ar/H_2 (90/10 vol%) between 200°C and 400°C within an alumina crucible placed in a quartz ampoule connected to a gas reservoir. Several heating and evacuating (dynamic vacuum) cycles were applied resulting in a fine U-powder.

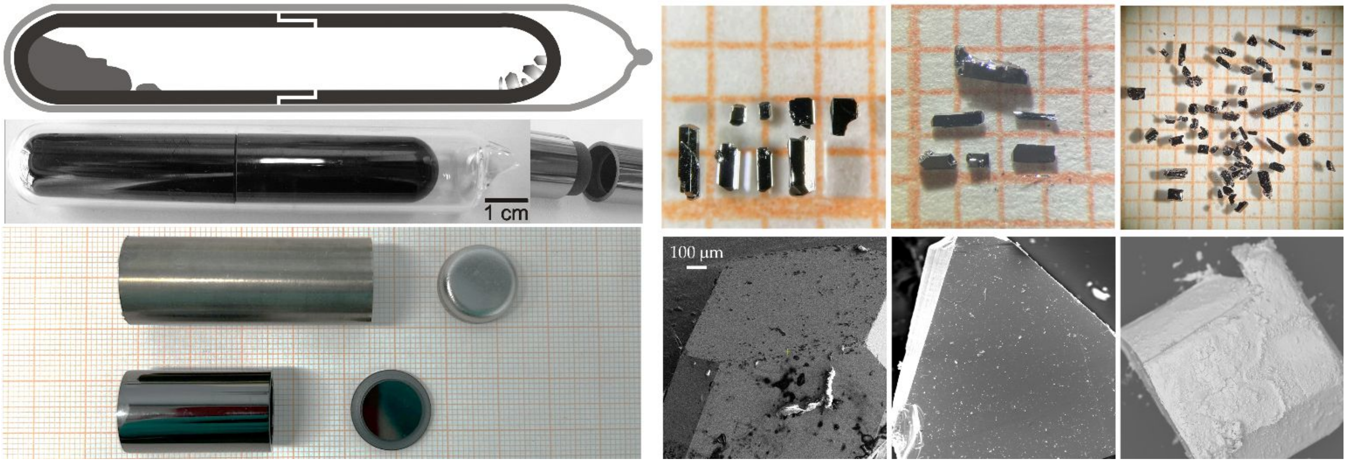

Figure S1. Top left: The set-up used for the oxygen-free CVT synthesis. A closed glassy carbon crucible prevents the direct contact of the sample with the quartz wall and thus formation of oxides. Bottom left: A glassy carbon inside a tantalum tube is used for the salt-flux synthesis of UTe_2. Top right: Single crystals of UTe_2 have similar appearance to the ones reported previously – dark metallic luster, mm-sized edges, rectangular shape. Bottom right: Scanning electron micrographs clearly show that the surfaces of these crystals are not perfect – some have residual flux, while others show signs of slight decomposition.

- Tellurium (Alfa Aesar 99.999%) was powdered in a glove box prior to use.
- Iodine (Alfa Aesar 99.998%) was supplied in thin quartz capillaries.
- The quartz ampoules were loaded in a glove box (O_2, H_2O < 0.1 ppm), sealed under dynamic vacuum (10^{-4} mbar) and placed in a furnace with a variable temperature gradient.
- UTe_2 crystallized by a chemical transport reaction in a temperature gradient with the mixture of the starting materials at the hot source and the formed crystals at the cool sink.

3. Salt-flux method (Samples 7, 8, and 9):

- First, uranium wire (natural uranium, Goodfellow, 99.9 %) was cleaned with HNO_3 for 10 sec and then rinsed with deionized water and acetone under a fume hood.
- The uranium wire, tellurium (Alfa Aesar, 99.999%), NaCl (Thermo Scientific Ultra Dry, 99.99%) and KCl (Thermo Scientific, 99.9995%) were used.
- The starting materials – 1-1.2 g of U and Te (ratio 1:2) and 2.5 g of salt mixture (ratio 1:1) were loaded in a glassy carbon crucible and enclosed in a Ta ampoule using the arc-melter.
- Ta ampule was placed in a vertical furnace and heated to 450°C in 24 h (dwelled for 24h), then heated to 950°C (dwelled for 24 h), cooled to 650°C over 192 h (dwelled for 24 h) and cooled down to room temperature over 48 h.
- Residual salt was washed away with water.

Sample characterization: The samples were characterized by powder X-ray diffraction (PXRD, with a LaB_6 standard), scanning electron microscopy (SEM), and transmission electron microscopy (TEM). The phase analysis was performed based on powder X-ray diffraction patterns (Huber Imaging Plate Guinier Camera G670, Cu Kα_1 radiation, λ = 1.540562 Å, LaB_6 (a = 4.15682(6) Å) as internal standard, WinXPow software). The lattice parameters were determined by a least-squares refinement using the peak positions, extracted by profile fitting using WinCSD.[4]

For metallographic investigations, the pieces of synthesized samples were embedded into polymer matrix using hot mounting press (ProntoPress 10). SiC paper and diamond powder with grain sizes of 3 µm or smaller were used for surface polishing. Preliminary, light microscopy (Axioplan 2, Zeiss) in bright field (estimation of phase distribution), dark field, polarized light (orientation and size of grains) and differential interference contrast (surface topography) was done. For elemental contrast as well as composition determination, scanning electron microscopy (SEM) using JEOL 7800 F with an attached EDX/EBSD system (Quantax 400, Bruker, Silicon-Drift-Detector (SDD)) was applied. For better spectral resolution and more accurate composition estimation, WDXS analysis (Cameca SX100 microprobe, tungsten electrode, acceleration voltage 20 kV) was carried out. As evident from Figure S2, for all investigated UTe_2 samples, the distribution of uranium and tellurium within the sample is rather homogeneous. However, while it was not possible to detect the presence of oxygen within the UTe_2, it is reasonable to assume that some inhomogeneities with respect to oxygen distribution do exist. These would certainly explain the change of the physical properties within some single crystals reported previously.[5]

Table S1: A summary of synthesis conditions and parameters for the UTe_2 single crystals studied in this work.

Sample number	Starting masses	Synthesis conditions	Lattice parameters[a]	Superconducting features[b]
1<br>red diamonds	0.4827 g U, 0.5174 g Te,<br>65 mg iodine,<br>U:Te ratio is 1:2	closed glassy carbon crucible, quartz ampoule, vacuum,<br>950°C-850°C gradient, 4 weeks	a = 4.1629(1) Å,<br>b = 6.1214(2) Å,<br>c = 13.9504(4) Å,<br>V = 355.49(2) Å	no superconductivity
		after 24 hours in air	a = 4.1630(1) Å,<br>b = 6.1216(2) Å,<br>c = 13.9512(4) Å,<br>V = 355.54(2) Å^3	no superconductivity
2<br>gray circles	0.4826 g U, 0.5115 g Te,<br>65 mg Iodine,<br>U:Te ratio is 1:1.98	open glassy carbon crucible, quartz ampoule, vacuum,<br>950°C-850°C gradient, 4 weeks	a = 4.1636(2) Å,<br>b = 6.1234(4) Å,<br>c = 13.9519(5) Å,<br>V = 355.71(3) Å^3	no superconductivity
3<br>yellow squares	0.5543 g U, 05393 g Te,<br>0.013 g TeO_2, 50 mg Iodine,<br>U:Te ratio is 1:1.81	quartz ampoule, vacuum,<br>950°C-860°C gradient, 4 weeks	a = 4.1611(2) Å,<br>b = 6.1320(2) Å,<br>c = 13.9719(5) Å,<br>V = 356.51(2) Å^3	two superconducting transitions:<br>T_{c2} = 1.50 K and T_c = 1.73 K
4<br>yellow hexagons	0.5579 g U, 0.44572 g Te,<br>25 mg Iodine, corresponds to 0.8 mg/cm3 in ampoule,<br>U:Te ratio is 1:1.49	quartz ampoule, vacuum,<br>875°C-775°C gradient, 2 weeks	a = 4.1628(2) Å,<br>b = 6.1344(4) Å,<br>c = 13.9770(7) Å,<br>V = 356.92(3) Å^3	two superconducting transitions:<br>T_c = 1.63 K and T_{c2} = 1.71 K
5<br>yellow triangles	0.5529 g U, 0.4466 g Te,<br>48 mg Iodine,<br>U:Te ratio is 1:1.51	open glassy carbon crucible, quartz ampoule, vacuum,<br>930°C-820°C gradient, 4 weeks	a = 4.1632(1) Å,<br>b = 6.1346(2) Å,<br>c = 13.9792(4) Å,<br>V = 357.02(2) Å^3	two superconducting transitions:<br>T_{c2} = 1.58 K and T_c = 1.72 K
6<br>orange stars	0.5622 g U, 0.4457g Te,<br>25 mg Iodine, corresponds to 0.8 mg/cm3 in ampoule,<br>U:Te ratio is 1:1.48	quartz ampoule, vacuum,<br>800°C-710°C gradient, 2 weeks	a = 4.1631(2) Å,<br>b = 6.1342(3) Å,<br>c = 13.9779(6) Å,<br>V = 356.96(3) Å^3	one superconducting transition:<br>T_c = 1.88 K
7<br>green pentagons	0.4826 g U, 0.5174 g Te,<br>NaCl 1.25 g, KCl 1.25 g,<br>U:Te ratio is 1:2	tantalum tube, glassy carbon crucible, argon atmosphere,<br>RT to 450 °C in 24 h, dwell for 24 h, ramp to 950 °C, dwell for 24 h,<br>cool to 650 °C in 192 h, dwell for 24 h,<br>cool to RT in 48 h	a = 4.1631(4) Å,<br>b = 6.1350(9) Å,<br>c = 13.9791(2) Å,<br>V = 357.03(5) Å^3	one superconducting transition:<br>T_c = 2.06 K
8<br>green diamonds	0.5791 g U, 0.6209 g Te,<br>NaCl 1.25 g, KCl 1.25 g,<br>U:Te ratio is 1:2		a = 4.1633(1) Å,<br>b = 6.1347(1) Å,<br>c = 13.9798(3) Å,<br>V = 357.05(1) Å^3	one superconducting transition:<br>T_c = 2.04 K
9<br>green triangles	0.5791 g U, 0.6209 g Te,<br>NaCl 1.25 g, KCl 1.25 g,<br>U:Te ratio is 1:2		a = 4.1636(1) Å,<br>b = 6.1350(1) Å,<br>c = 13.9791(3) Å,<br>V = 357.08(1) Å^3	one superconducting transition:<br>T_c = 2.08 K

[a] The lattice parameters were obtained from the powder diffraction pattern recorded with CuK~\a1~ radiation by using LaB_6 as internal standard (a = 4.15692 Å).
[b] Similar to previous reports, the value of superconducting temperature T_c is taken as the midpoint of the rise in C/T.

Table S2: Crystallographic information and refinement results for UTe_2 single crystals[a]

	Sample 1	Sample 2	Sample 3	Sample 4	Sample 5	Sample 6	Sample 7	Sample 8	Sample 9
Uranium occupancy	0.947(4)	0.959(4)	1.002(6)	0.996(5)	1.002(6)	0.998(6)	1.004(10)	0.999(5)	0.993(5)
Structure type	UTe_2								
Space group	*Immm* (No 71)								
Z	4								
Calc. density / g cm−1	9.214	9.032	9.189	9.179	9.175	9.225	9.174	9.175	9.174
Range in *h*, *k*, *l*	$-4 \le h \le 7$	$-7 \le h \le 6$	$-7 \le h \le 6$	$-4 \le h \le 7$	$-7 \le h \le 7$	$-8 \le h \le 4$	$-7 \le h \le 7$	$-7 \le h \le 5$	$-5 \le h \le 7$
	$-11 \le k \le 11$	$-9 \le k \le 11$	$-4 \le k \le 11$	$-9 \le k \le 11$	$-7 \le k \le 11$	$-11 \le k \le 8$	$-8 \le k \le 11$	$-7 \le k \le 11$	$-10 \le k \le 11$
	$-26 \le l \le 16$	$-14 \le l \le 26$	$-11 \le l \le 25$	$-21 \le l \le 26$	$-14 \le l \le 26$	$-26 \le l \le 23$	$-13 \le l \le 25$	$-20 \le l \le 25$	$-14 \le l \le 26$
Absorption coeff. / mm^{-1}	61.38	59.56	61.25	60.10	61.12	61.08	61.21	61.15	61.12
N(*hkl*) measured	3637	3670	3683	3260	3781	3722	3461	3350	3860
N(*hkl*) unique	765	771	765	696	771	702	660	698	771
N(*hkl*) observed	747	744	729	669	747	644	645	693	759
Refined parameters	14	15	14	14	14	15	14	14	14
R_F	0.0324	0.0252	0.0284	0.0259	0.0340	0.0381	0.0359	0.0277	0.0273
R_W	0.0346	0.0360	0.0293	0.0272	0.0364	0.0408	0.0383	0.0294	0.0285
Residual peaks / e $Å^{-3}$	-3.040 / 2.601	-2.627 / 2.745	-6.658 / 3.651	-2.001 / 5.959	-3.730 / 3.704	-3.537 / 4.254	-6.511 / 4.206	-3.253 / 3.689	-4.867 / 3.231
CCDC number	2431421	2431402	2431420	2474922	2431423	2474914	2431422	2431425	2431426

[a] In the final runs of the refinements the lattice parameters obtained from powder diffraction data were used (see Table S1)

Table S3: Crystallographic information and refinement results for UTe_2 single crystals.

		Sample 1	Sample 2	Sample 3	Sample 4	Sample 5	Sample 6	Sample 7	Sample 8	Sample 9
U in 4i position	x/a	0	0	0	0	0	0	0	0	0
	y/b	0	0	0	0	0	0	0	0	0
	z/c	0.36443(3)	0.36448(2)	0.36487(3)	0.36486(3)	0.36485(3)	0.36486(4)	0.36483(9)	0.36485(3)	0.36485(6)
	$U_{eq./iso}$	0.0109(1)	0.00946(8)	0.00733(10)	0.00754(9)	0.0091(1)	0.0107(1)	0.00973(13)	0.0100(1)	0.00860(8)
	U_{11}	0.0107(2)	0.0088(2)	0.0071(2	0.0058(2)	0.0090(2)	0.0089(2)	0.01190(18)	0.0094(2)	0.0079(2)
	U_{22}	0.0109(2)	0.0096(2)	0.0074(2)	0.0083(3)	0.0094(2)	0.0117(2)	0.00948(19)	0.0110(2)	0.0092(2)
	U_{33}	0.0112(2)	0.0100(2)	0.0074(2)	0.0084(2)	0.0090(2)	0.0117(2)	0.00783(18)	0.0095(2)	0.0087(2)
Te1 in 4g position	x/a	0	0	0	0	0	0	0	0	0
	y/b	0.2510(1)	0.25103(8)	0.2510(1)	0.2510(1)	0.2508(1)	0.2506(2)	0.2509(2)	0.2510(1)	0.25082(9)
	z/c	0	0	0	0	0	0	0	0	0
	$U_{eq./iso}$	0.0096(2)	0.0093(1)	0.0076(2)	0.0076(2)	0.0090(2)	0.0103(2)	0.00999(15)	0.0100(2)	0.0087(1)
	U_{11}	0.0106(3)	0.0099(2)	0.0080(3)	0.0066(3)	0.0091(3)	0.0087(4)	0.0132(3)	0.0099(3)	0.0086(2)
	U_{22}	0.0082(3)	0.0080(2)	0.0064(3)	0.0071(2)	0.0083(3)	0.0101(4)	0.0086(3)	0.0099(3)	0.0081(2)
	U_{33}	0.0101(3)	0.0101(2)	0.0084(3)	0.0092(3)	0.0095(3)	0.0120(4)	0.0082(3)	0.0103(3)	0.0095(2)
Te2 in 4j position	x/a	½	½	½	½	½	½	½	½	½
	y/b	0	0	0	0	0	0	0	0	0
	z/c	0.20259(5)	0.20248(4)	0.20210(5)	0.20215(5)	0.20223(6)	0.20210(7)	0.20223(7)	0.20213(5)	0.20211(4)
	$U_{eq./iso}$	0.0102(2)	0.0100(1)	0.0080(2)	0.0080(2)	0.0093(2)	0.0108(2)	0.00975(15)	0.0104(2)	0.0090(1)
	U_{11}	0.0118(3)	0.0111(2)	0.0092(3)	0.0076(3)	0.0106(3)	0.0107(4)	0.0121(3)	0.0110(3)	0.0094(2)
	U_{22}	0.0084(3)	0.0087(2)	0.0069(3)	0.0075(2)	0.0081(3)	0.0102(4)	0.0083(3)	0.0103(3)	0.0085(2)
	U_{33}	0.0104(3)	0.0102(2)	0.0080(3)	0.0089(3)	0.0092(3)	0.0114(4)	0.0089(3)	0.0099(3)	0.0092(2)

For each atomic position $U_{12} = U_{13} = U_{23} = 0$

Transmission electron microscopy analysis: Atomic-resolution imaging by high-angle annular dark-field (HAADF) scanning transmission electron microscopy (STEM) was performed on a double aberration-corrected JEOL GrandARM S/TEM operating at 300 kV with a probe convergence semi-angle of 25 mrad and inner (outer) collection angles of 68 (280) mrad. Low-angle annular dark-field (LAADF)-STEM imaging over large areas used inner (outer) collection angles of 27 (110) mrad. Cross-sectional STEM specimens were prepared by the standard focused-ion-beam (FIB) lift-out procedure and coordinated to reduce the amount of air exposure during transfer between the inert glovebox atmosphere, FIB, and STEM as much as possible such that the total air exposure of the thinned lamella is less than five minutes from the FIB to the STEM airlock.

Physical properties measurements: Specific heat was measured down to 0.4 K on single crystals of UTe_2 in a Quantum Design (QD) Physical Property Measurement System (PPMS). In order to ensure that the samples are protected from decomposition, all handling was done inside a glovebox. The specific heat data is consistent with what has been reported for previous samples of UTe_2. In particular, for the UTe_2 with the sharpest superconducting anomaly, the extracted parameters ($\Delta C_e/(\gamma T_c)$ = 2.2, γ_{SC} = 10.1 mJ mol^{-1} K^{-2} and γ_{SC}/γ_N = 0.09) are consistent with what is currently considered to be "state-of-the-art" UTe_2 samples.

Micro-scale isolation: In order to eliminate possible sample-to-sample variation, specific heat and single crystal analysis were carried out on the same exact crystals of UTe_2. First, specific heat data were collected, as described above. Then, micro-scale specimens (~30x30x30 μm3) were extracted using a Xe-plasma focused-ion-beam (FEI Helios G4 PFIB. Details of this method are similar to what has previously been done for other strongly correlated systems.[6–8] For some of the crystals, specimens were extracted from both inner and outer parts, in order to examine the effect of air-sensitivity of UTe_2.

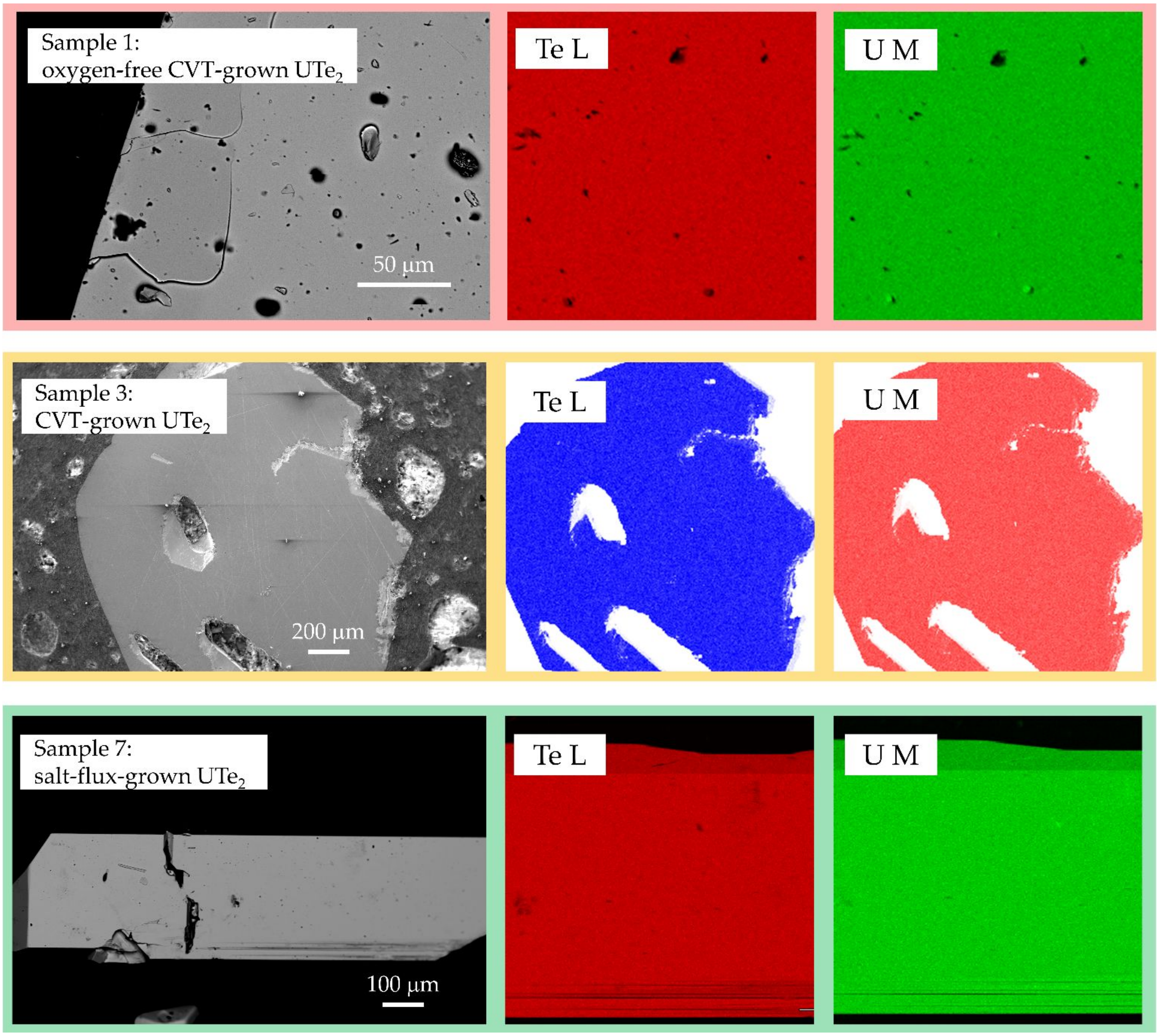

Figure S2. Scanning electron micrographs for UTe_2 grown via oxygen-free-CVT method (top, Sample 1, no superconductivity), traditional CVT method (top, Sample 3, two superconducting transitions), and salf-flux method (bottom, Sample 7, one superconducting transition). For all samples, distribution of both uranium and tellurium is homogeneous within the instrumental resolution.

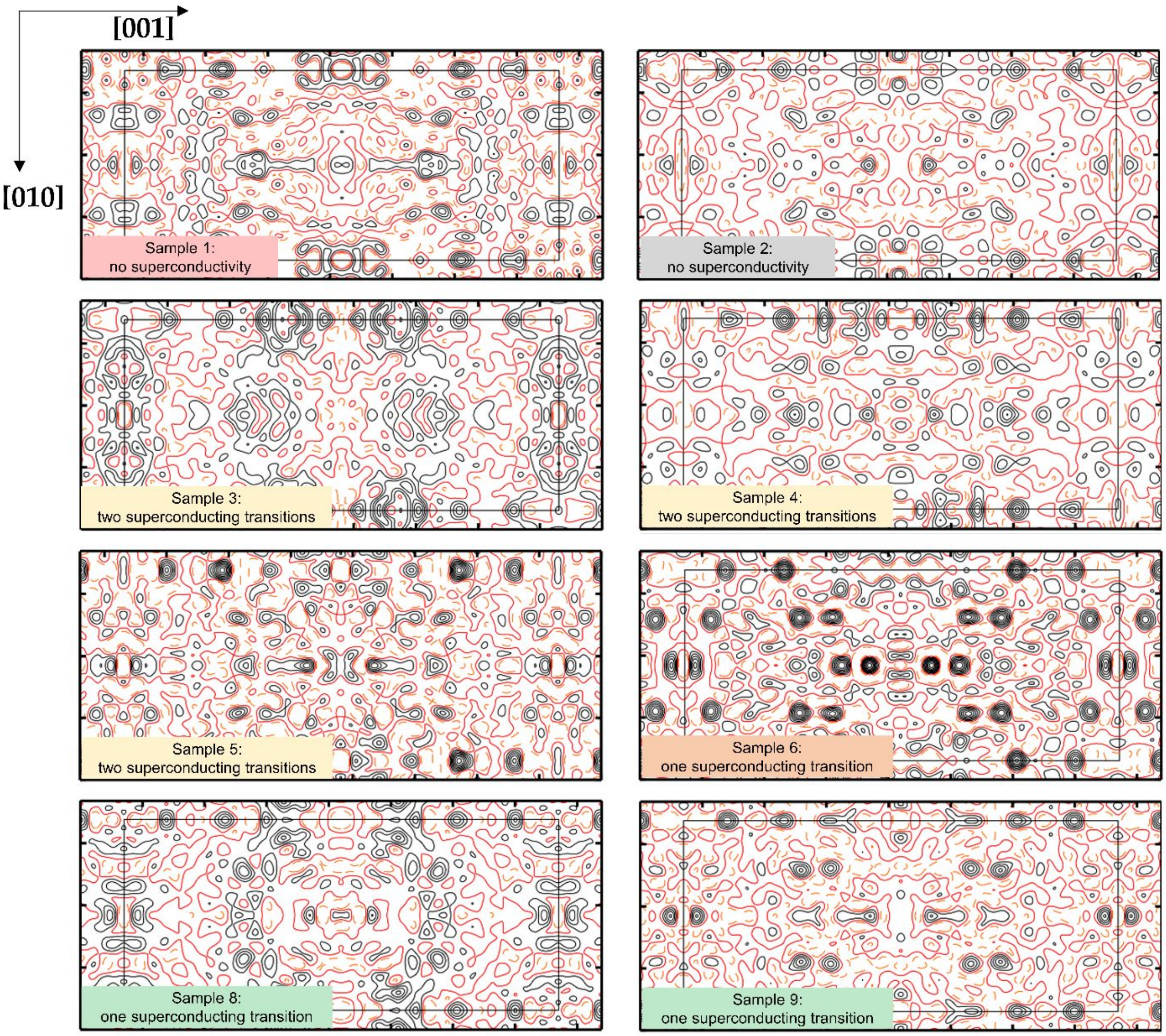

Figure S3. Difference electron density maps of eight UTe_2 samples in the (100) plane after refinement of the ordered UTe_2 atomic arrangement. Black rectangle shows the borders of the unit cell. The isolines are drawn with the step of 1 e/Å^3 (positive values are shown in black, zero – in red solid lines, negative – in orange dashed lines). For ease of viewing, only the first negative iso-value is shown. The scale on the axes has an increment of 1 Å (black tick marks).

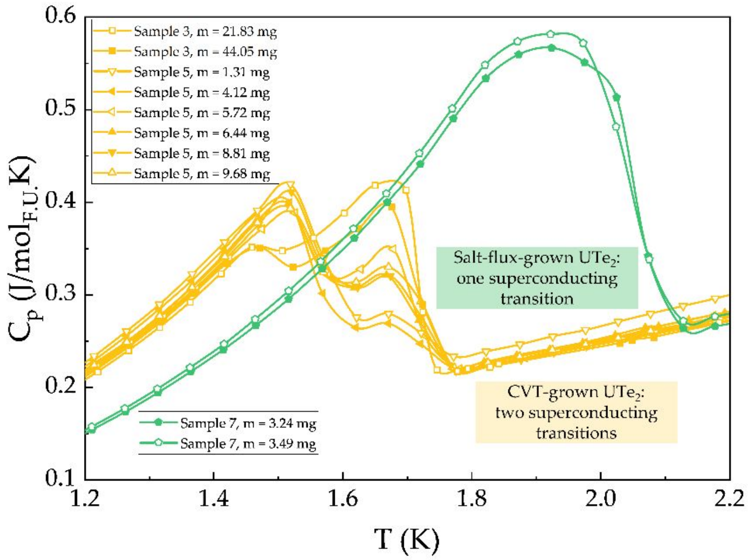

Figure S4. Specific heat anomaly corresponding to the entrance into superconducting state. For Samples 3 and 5, two transitions are observed, with some crystal-to-crystal variation of the respective peak heights. For Sample 7, the position and shape of the transition are virtually the same.

References:

(1) Leithe-Jasper, A.; Borrmann, H.; Hönle, W. *MPI CPfS, Scientific Report*; Dresden, 2005.

(2) Svanidze, E.; Amon, A.; Gumeniuk, R.; Leithe-jasper, A. Intermetallic Compounds with Thorium and Uranium. *MPI CPfS Status Report* 2018.

(3) Harris, I. R.; Noble, C.; Bailey, T. The Hydrogen Decrepitation of an $Nd_{15}Fe_{77}B_8$ Magnetic Alloy. *Journal of The Less-Common Metals* 1985, *106* (1). https://doi.org/10.1016/0022-5088(85)90380-7.

(4) Akselrud, L.; Grin, Y. WinCSD: Software Package for Crystallographic Calculations (Version 4). *J. Appl. Crystallogr.* 2014, *47* (2), 803–805. https://doi.org/10.1107/S1600576714001058.

(5) Thomas, S. M.; Stevens, C.; Santos, F. B.; Fender, S. S.; Bauer, E. D.; Ronning, F.; Thompson, J. D.; Huxley, A.; Rosa, P. F. S. Spatially Inhomogeneous Superconductivity in UTe_2. *Phys. Rev. B* 2021, *104* (22), 224501. https://doi.org/10.1103/PhysRevB.104.224501.

(6) Moll, P. J. W. Focused Ion Beam Microstructuring of Quantum Matter. *Annu. Rev. Condens. Matter Phys.* 2018, *9* (1), 147–162. https://doi.org/10.1146/annurev-conmatphys-033117-054021.

(7) Antonyshyn, I.; Wagner, F. R.; Bobnar, M.; Sichevych, O.; Burkhardt, U.; Schmidt, M.; König, M.; Poeppelmeier, K.; Mackenzie, A. P.; Svanidze, E.; Grin, Y. Micro-Scale Device—An Alternative Route for Studying the Intrinsic Properties of Solid-State Materials: The Case of Semiconducting TaGeIr. *Angew. Chem.* 2020, *59* (27), 11136–11141. https://doi.org/10.1002/anie.202002693.

(8) Amon, A.; Svanidze, E.; Ormeci, A.; König, M.; Kasinathan, D.; Takegami, D.; Prots, Y.; Liao, Y. F.; Tsuei, K. D.; Tjeng, L. H.; Leithe-Jasper, A.; Grin, Y. Interplay of Atomic Interactions in the Intermetallic Semiconductor Be_5Pt. *Angew. Chem.* 2019, *58* (44), 15928–15933. https://doi.org/10.1002/anie.201909782.
